# Editorial: COVID-19 and Women's Health

**DOI:** 10.3389/fgwh.2022.861315

**Published:** 2022-03-03

**Authors:** Laura A. Magee, Vassiliki Benetou, Rhiannon George-Carey, Jayashri Kulkarni, Nathalie Emma MacDermott, Stacey A. Missmer, Chelsea Morroni, Marianne Vidler, Stephen H. Kennedy

**Affiliations:** ^1^Institute of Women and Children's Health, King's College London, London, United Kingdom; ^2^Department of Hygiene, Epidemiology and Medical Statistics, National and Kapodistrian University of Athens, Athens, Greece; ^3^North Middlesex University Hospital NHS Trust, London, United Kingdom; ^4^Monash University, Melbourne, VIC, Australia; ^5^Department of Women and Children's Health, King's College London, London, United Kingdom; ^6^College of Human Medicine, Michigan State University, East Lansing, MI, United States; ^7^MRC Center for Reproductive Health, University of Edinburgh, Edinburgh, United Kingdom; ^8^Botswana Harvard AIDS Institute Partnership, Gaborone, Botswana; ^9^Department of Obstetrics and Gynaecology, University of British Columbia, Vancouver, BC, Canada; ^10^Nuffield Department of Women's and Reproductive Health, University of Oxford, Oxford, United Kingdom

**Keywords:** women's health, COVID-19, pregnancy, pandemic consequences, gender equality in health

## Introduction

The SARS-CoV-2 virus that causes COVID-19 emerged in Wuhan, China, in November 2019. Cases were officially recognized in December 2019 and, by February 2020, the virus had spread internationally. On 11 March 2020, the World Health Organization declared the outbreak a global pandemic.

COVID-19 has had a profoundly negative impact globally on physical and mental health, health care, and social functioning, across all age groups and medical conditions, although women may have been disproportionately affected.

What seemed initially to require a brief period of global lockdown, with at worst a return to the delivery of normal health services by autumn 2020, has resulted worldwide in ongoing restrictions in social activity and travel despite successful international vaccination programmes and infectious disease tracking and tracing.

Our Research Topic was launched on 5 May 2020 with a planned close on 8 September 2020, which was extended until 31 December 2020 due to a high number of submissions. These reflect people's experiences and associated health outcomes during the first and second waves of COVID-19.

## Outline of Contributions

Our Research Topic attracted keen interest from potential contributors worldwide. We accepted 39 manuscripts from the Americas (*N* = 18), Australasia (*N* = 2), Europe (*N* = 12), Africa (*N* = 2), and Asia (*N* = 5), covering a broad range of methodologies: a brief research report (*N* = 1), case reports/studies (*N* = 2), mini-reviews (*N* = 5), opinions (*N* = 6), original research (*N* = 12), perspectives (*N* = 5), reviews (*N* = 5), and systematic reviews (*N* = 3). Most of the manuscripts were in the fields of maternal health (*N* = 18) or maternal mental health (*N* = 14). Our comments below focus on the five top-viewed and downloaded papers we published.

By September 2020, a rapid narrative review by Mittal and Singh was published concerning gender-based violence during both current and prior pandemics (200,486 views, 7,699 downloads). Authors focused on the first wave of the pandemic and use of the quarantine as the primary measure to reduce disease spread, before the emergence of an effective vaccine. There was an alarming rise in gender-based violence, with risk factors including economic insecurity and alcohol consumption. Many services remained inadequate, as they had been pre-pandemic, and importantly, women remained both more disconnected from those services and from their prior support networks. A sobering aspect to this review was the observation that all of this has been observed before, as disruption of social norms tends to increase violence and gender-based violence, specifically. Interestingly, the overwhelming majority of people who viewed this paper reside in South Africa.

Davenport et al. (95,203 views, 8,288 downloads) undertook a rapid online survey via social media platforms of 900 pregnant or postpartum women in the earliest stages of the pandemic (i.e., April and May 2020), focusing on mental health and physical activity. Most women were from Canada, White, married, living in a single-family home, and had some post-secondary education. The authors documented a substantial increase in self-reported depression and anxiety, compared with pre-pandemic levels, recorded by validated screening questionnaires of depression/depressive symptoms. While two-thirds of women reduced their physical activity, 15% increased it, and those engaging in at least 150 mins per week of moderate-intensity physical activity, consistent with current physical activity guidelines, had significantly lower depression and anxiety scores—a potentially empowering message.

In their review, Thibaut and van Wijngaarden-Cremers (32,113 views, 5,458 downloads) showed that the pandemic was affecting the mental health of women more profoundly than men, as frontline workers, especially in the health and social sector, and as the primary care-givers in the home. There were echoes of financial challenges (including a higher likelihood of extreme poverty for young women) and mental health problems growing, as highlighted by the earlier published work of these authors and others. Importantly, the opportunity for positive change was emphasized, recognizing the major role of women at home and in the workplace; we were reminded of the example of the post-World War II era in which gender equality improved at home and in the workplace, and society became more resilient.^1^

When the pandemic first emerged, there was concern that pregnant women would be more susceptible to SARS-CoV-2 infection, as they had been with other coronavirus outbreaks, i.e., Severe Acute Respiratory Syndrome (SARS) and Middle East Respiratory Syndrome (MERS). Although by February 2021, this was no longer thought to be the case, it was clear that when infected, pregnant women were more likely to develop severe disease and pregnancy complications (particularly preterm birth), especially in the third trimester. The Vale et al. review (18,777 views, 902 downloads) explored why this is the case. In brief, pregnancy is associated with physiological changes in the respiratory system—notably, vascular congestion and oedema of the upper respiratory tract, and a reduced expiratory reserve volume due to a raised diaphragm. Also, pregnancy is associated with immune adaptations, required to tolerate the fetus' paternal antigens, whilst preserving an adequate immune response against invading microorganisms. Of note, there is a natural increase in pro-inflammatory mediators in the first and third trimesters, that may augment the “cytokine storm” associated with COVID-19, leading to more severe disease. More changes during pregnancy that make pregnant women more susceptible to SARS-Cov-2 infection, in addition to an increased risk of developing more severe disease, are presented in this review.

While COVID-19 is clearly associated with more pregnancy complications, it is also clear that the pandemic itself has disrupted maternity services for all pregnant women, whether infected or not. This was highlighted by Oluoch-Aridi et al. (9,399 views, 1,338 downloads) through qualitative interviews in our fifth most viewed and downloaded publication. While the setting was informal settlements in Nairobi, Kenya, the messages are widely applicable to other health care settings. There was evidence that fear of infection was a barrier to care-seeking, but so was financial hardship a barrier to transportation. There were some improvements in quality of care, particularly outpatient care, in terms of shorter waiting times, as well as better hygiene measures and more responsive health personnel. However, the prohibition of friends and family accompanying women to health facilities was described as disrespectful, a breach of ethical guidelines, and a frank violation of human rights.

## Concluding Remarks

Our Research Topic attracted a broad range of manuscripts from researchers across the globe, with more than half of studies focussed on women's health in general (rather than pregnancy/postpartum specifically), and as much interest in the indirect consequences of the pandemic as in the direct implications of SARS-CoV-2 infection itself. This represents a broad collection of global perspectives. The topic dovetails with two others. First, is the “Vaccination in pregnancy” topic that loses for submission of abstracts on 20 January 2022, and to full manuscripts on 31 January 2022 (https://www.frontiersin.org/research-topics/25767/vaccination-in-pregnancy). Second, is “SDG5 in a Post-COVID World -Achieving Gender Equity in Health,” that closes for submission of abstracts on 08 February 2022, and to full manuscripts on 09 April 2022 (link to be added w/c Jan 31st). (https://www.frontiersin.org/research-topics/32121/sdg5-in-a-post-covid-world-achieving-gender-equity-in-health).

## Author Contributions

SK and LM outlined the general structure of the editorial. LM wrote the initial draft. All authors contributed to the article and approved the submitted version.

## Conflict of Interest

The authors declare that the research was conducted in the absence of any commercial or financial relationships that could be construed as a potential conflict of interest.

## Publisher's Note

All claims expressed in this article are solely those of the authors and do not necessarily represent those of their affiliated organizations, or those of the publisher, the editors and the reviewers. Any product that may be evaluated in this article, or claim that may be made by its manufacturer, is not guaranteed or endorsed by the publisher.

## Footnotes

^1^ https://www.theguardian.com/lifeandstyle/2018/feb/03/1940s-britains-wartime-women-gained-a-new-sense-of-power

